# Real-time PCR analysis of a 3895 bp mitochondrial DNA deletion in nonmelanoma skin cancer and its use as a quantitative marker for sunlight exposure in human skin

**DOI:** 10.1038/sj.bjc.6603178

**Published:** 2006-05-23

**Authors:** A Harbottle, M A Birch-Machin

**Affiliations:** 1 Dermatological Sciences, School of Clinical and Laboratory Sciences, University of Newcastle, Newcastle upon Tyne NE2 4HH, UK

**Keywords:** mitochondrial DNA, skin cancer, ultraviolet radiation, DNA deletion, real-time PCR

## Abstract

Previous findings from our own laboratory have shown that the frequency of occurrence (i.e. the simple presence or absence) of the 3895 bp mitochondrial DNA deletion is increased with increasing sun exposure. The present study has significantly extended this work by developing, validating and then using a quantitative real-time PCR assay to investigate for the first time the actual level (as opposed to the frequency of occurrence) of the 3895 bp deletion in human skin from different sun-exposed body sites and tumours from nonmelanoma skin cancer patients. We investigated the 3895 bp deletion in 104 age-matched split human skin samples taken from various sun-exposed sites defined as usually exposed (*n*=60) and occasionally exposed (*n*=44) when outdoors. The results clearly show an increased level of the 3895 bp deletion with increasing sun exposure. Specifically, there was a significantly higher level of the deletion in the usually sun-exposed compared to the occasionally sun-exposed skin (*P*=0.0009 for dermis, *P*=0.008 for epidermis; two-tailed *t*-test). Our study has also extended previous findings by showing that the level of the 3895 bp deletion is significantly higher in the dermis compared with the epidermis both in the occasionally sun-exposed samples (*P*=0.0143) and in the usually sun-exposed skin. (*P*=0.0007).

The incidence of nonmelanoma skin cancer (NMSC) is increasing in populations of European origin (Severi and English, 2004), for example, 1 million new cases diagnosed each year in the USA (Wesson and Silverberg, 2003) and 75 000 in the UK (figures provided by Cancer Research UK). Nonmelanoma skin cancer accounts for around 90% of skin cancers and consists of basal cell and squamous cell carcinomas (BCC and SCC, respectively). Ultraviolet radiation (UVR) is the major determinant of NMSC. It is a significant factor in the generation of oxidative damage, which can lead to DNA strand breaks and to the production of DNA photoproducts such as thymidine dimers, which importantly cannot be repaired in mitochondrial DNA (mtDNA) (Clayton *et al*, 1974). In addition mtDNA is located in the matrix, which is in close proximity to the inner membrane where reactive oxygen species (ROS) are continually produced in the electron transport chain. This along with the absence of protective histones makes mtDNA a sensitive marker of UV-induced DNA damage compared to nuclear DNA (LeDoux *et al*, 1993; Croteau and Bohr, 1997; Pascucci *et al*, 1997; Sawyer and Van Houten, 1999; Birch-Machin, 2000). Finally, each cell can contain up to several thousand copies of the mtDNA genome and mitochondria can, therefore, tolerate very high levels (up to 90%) damaged mtDNA through complementation of the remaining wild-type (Chomyn *et al*, 1992; Sciacco *et al*, 1994). Therefore, cells are able to accumulate photodamage in mtDNA without compromising cell function.

To determine a reliable marker of cumulative UVR exposure in human skin, our research group and others have examined the novel idea of using mtDNA, rather than nuclear DNA, as a biomarker of UV-induced DNA damage (Pang *et al*, 1994; Berneburg *et al*, 1997; Birch-Machin *et al*, 1998; Birch-Machin, 2000). Furthermore, we and others have shown that mtDNA deletions can be induced in cultured human skin cells by sub-lethal repetitive doses of UVA (Berneburg *et al*, 1999; Krishnan *et al*, 2004). In agreement with observations of mtDNA damage in other tumours (Birch-Machin, 2006; Czarnecka *et al*, 2006; Parr *et al*, 2006) we have previously identified somatic mtDNA changes in human skin as a result of the first detailed study of mtDNA damage in NMSC (Durham *et al*, 2002). Importantly, as a result of this study, we identified a 3895 bp mtDNA deletion which occurred more frequently in usually sun-exposed skin as opposed to occasionally sun-exposed skin (Harbottle *et al*, 2004; Krishnan *et al*, 2004). This is important because the relative density of NMSC is highest on body sites ‘usually’ exposed to the sun when outdoors (such as scalp, face, neck and ears as defined by Armstrong, 2004) compared to occasionally sun-exposed sites (such as shoulders, back and chest as defined by Armstrong, 2004).

Our previous study (Krishnan *et al*, 2004) showed that the frequency of occurrence of the 3895 bp deletion in human skin provides a potential biomarker for cumulative UV exposure in human skin. The study also claimed that possible applications of this biomarker may in turn provide an early detection tool for NMSC development as well as providing a method of monitoring long-term safety of clinical UV phototherapy regimes. However, in order for this type of study to progress and become a reality one needs to be able to quantify the level of the 3895 bp deletion rather than simply determine the frequency of occurrence (i.e. presence or absence) of the deletion as reported in our previous study (Krishnan *et al*, 2004). This has relevance to mitochondrial function because the regions that are deleted in the 3895 bp deletion are functionally important. The deleted region includes the mtTF1 binding site in the D-loop to tRNA methionine and deleted genes include 12 s rRNA, 16 s rRNA, ND1 as well as the promoters for transcription of both the H and L strands. There are several key questions, which remain unanswered by our previous study. One notable question is whether the greater frequency of occurrence in the dermis *vs* the epidermis is mirrored by a greater actual level of damage in the dermis when mtDNA damage is also present in the corresponding epidermis. A second important question is whether there is a greater absolute level of the 3895 bp deletion in usually sun-exposed compared to occasionally sun-exposed skin in addition to the greater frequency of occurrence observed in our previous study. The present study seeks to overcome this clear limitation by developing a quantitative and reliable real-time PCR assay for the 3895 bp deletion. Levels of the 3895 bp deletion are then determined in tumour and perilesional skin taken from NMSC patients as well as skin (including both epidermis and dermis) taken from different sun-exposed body sites.

## MATERIALS AND METHODS

### Human skin samples

Tumour and matched perilesional skin samples were taken with informed consent from patients undergoing excision of a NMSC, namely basal cell carcinoma (BCC) (*n*=5, age range 55–89 years (yrs), mean 78 yrs) or a squamous cell carcinoma (SCC) (*n*=5, age range 70–87 yrs, mean 78 yrs) at the Outpatients Clinic, Royal Victoria Infirmary, Newcastle, UK. For the sun exposure studies, clinically normal perilesional skin was taken from body sites which are ‘usually’ exposed to the sun when outdoors (such as scalp, face, neck and ears) (epidermis *n*=30, dermis *n*=30, mean age ±s.e.m.=70.45±2.161) and body sites which are ‘occasionally’ exposed to the sun (shoulders, back and chest) (epidermis *n*=22, dermis *n*=22, mean age ±s.e.m.=63.77±3.501). There are no significant age differences between the usually and occasionally sun-exposed groups (*P*=0.1134): two-tailed *t*-test (Welch correction). For all perilesional skin samples, epidermis and dermis were separated using 0.25% dispase at 4°C overnight (Durham *et al*, 2003) and DNA was extracted using a Qiagen, DNeasy tissue extraction kit. None of the patients used for this study suffered from a mitochondrial myopathy or had a mtDNA associated neurodegenerative disease.

### PCR analysis

**Figure 1:**
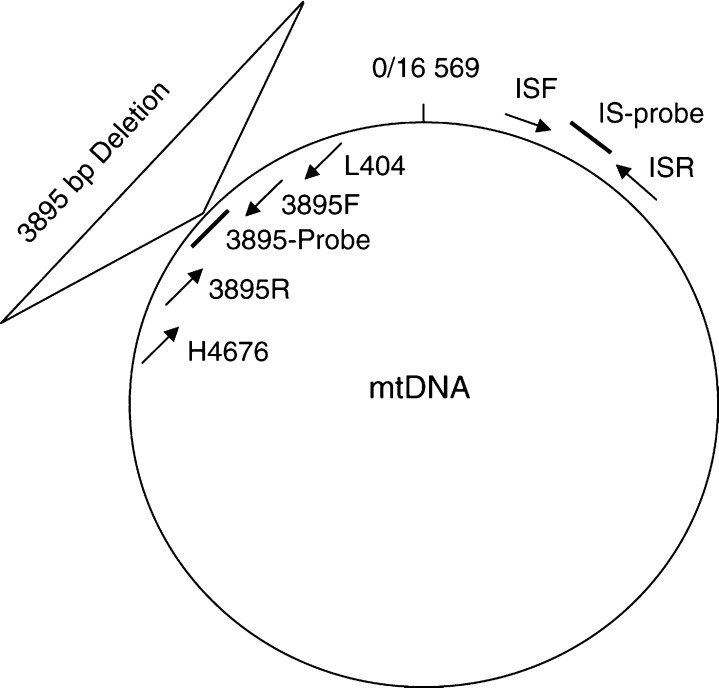
Localisation of PCR primers and TaqMan probes. Schematic representation of the mtDNA genome containing the 3895 bp deletion. Primers ISF/ISR and IS Probe, anneals to both wild-type and deleted mtDNA. Detection of the 3895 bp deletion was performed with primers 3895F/3895R and 3895 Probe. The specific 3895 Probe only anneals to deleted mtDNA as it binds across the deletion junction. In addition, the occurence of the 3895 bp deletion brings together the deletion specific primers (i.e. 3895F:3895R and L404:H4676) close enough to allow generation of an amplicon under the given PCR conditions. The deleted region includes the mtTF1 binding site in the D-loop to tRNA methionine and deleted genes include 12 s rRNA, 16 s rRNA, ND1 as well as the promoters for transcription of both the H and L strands.

The PCR was carried out in a 25 *μ*l reaction containing 200 ng genomic DNA, 600 nM of each primer, 250 *μ*M dNTPs, 0.6 U per reaction Amplitaq Gold DNA polymerase (Applied Biosystems, Warrington, UK), GeneAmp buffer (containing, 100 mM Tris-HCl, pH 8.3, 500 mM KCl, 15 mM MgCl and 0.01% (w v^−1^) gelatin). The PCR primers L404 and H4676 (Table 1 and Figure 1) were designed to anneal outside the 3895 bp deletion. During DNA amplification the short (30 s) polymerase extension time did not permit amplification of wild-type PCR products, allowing only amplification of the shorter and deleted mtDNA fragments. The PCR conditions were 94°C for 10 min, 35 cycles of 94°C for 30 s, 56°C for 30 s, 72°C for 30 s and a final extension of 7 min at 72°C. Amplification products were visualised in a 1% agarose gel stained with ethidium bromide (0.25 *μ*g ml^−1^).

### Real-time PCR analysis

We have developed and established a reliable TaqMan-PCR assay for the quantification of the 3895 bp deletion. The quantitative TaqMan-PCR method provides real-time measurement of target input as PCR accumulation through a dual labelled probe. The probe anneals between forward and reverse primers and it is cleaved by the 5′–3′ exonuclease activity of Taq polymerase during the PCR extension phase. Therefore, the 5′-terminal reporter dye FAM (6-carboxyfluorescein) or VIC and the 3′-terminal quencher dye TAMRA (6-carboxy-*N*,*N*,*N*′,*N*′-tetramethylrhodamine) linked to the probe are separated, resulting in a fluorescence emission of the reporter dye. The probe is not able to serve as a primer itself because it is 3′-terminally blocked with a phosphate group. The method uses an internal standard probe (IS-Probe, Table 1 and Figure 1) in the cytochrome *b* region of the genome (Koch *et al*, 2001), to estimate total copy number for mtDNA (i.e. deleted and wild type). The level of 3895 bp deletion is determined by a probe (3895-probe, Table 1 and Figure 1) which spans the break point of the deletion ensuring that it is only amplified if the deletion is present. Quantification of the level of deletion is determined by comparison of the ratio of the internal standard to the 3895 bp deletion.

Amplification reactions were performed as 25 *μ*l triplicates in a 96-well microplate. Total mtDNA and deleted mtDNA reactions were amplified in separate tubes, each containing 100 ng of DNA, 1 × TaqMan Universal Mastermix (ABI), 300 nM of each internal standard primer (ISF and ISR, Table 1 and Figure 1) and 100 nM of the IS-Probe, or 300 nM of each 3895 bp deletion primer (3895F and 3895R, Table 1) and 100 nM of the 3895-probe (Figure 1). PCR and fluorescence analysis was performed using a ABI Prism 7000 (Applied Biosystems, UK). Amplification conditions were: 2 min at 50°C, 10 min at 95°C followed by 40 cycles of 15 s at 95°C and 1 min at 60°C. The *R*_n_ value is the ratio of the emission intensity of the passive reference, a dye included in the TaqMan reaction buffer. Δ*R*_n_ is defined as the difference between *R*_n_^+^ (*R*_n_ of a reaction containing all the components including template) and *R*_n_^−^ (*R*_n_ of a no template control). The cycle at which a statistically significant increase in Δ*R*_n_ is detected first is called the threshold cycle (*C*_t_). Fluorescence signals are regarded as significant if the fluorescent intensity exceeds 10-fold the s.d. of the background *R*_n_ value to define a threshold.

Absolute DNA quantification was performed using the standard curve method. Reactions were carried out with different concentrations of two standard plasmids (for cloning see section below) in parallel to the samples that should be quantified. The standard plasmids, one carrying sequences flanking the 3895 bp deletion (i.e. the region covered by the 3895F and R primers) and one carrying a unique sequence of the mtDNA independent of the 3895 bp deletion (i.e. the region covered by the ISF and R primers), allowed the generation of two standard curves showing the number of copies of total mtDNA or mtDNA harbouring the 3895 bp deletion *vs* the measured *C*_t_. The *C*_t_ values of samples could then easily be converted to the number of DNA copies by comparing *C*_t_ for the sample to the *Ct* for the respective standard plasmid of known concentrations. The concentration of each template was determined fluorometrically (Turner Biosytems, UK). The amount of mutation corresponds to the ratio of mtDNA with the 3895 bp deletion to wild-type mtDNA within each sample. If not detected within 40 cycles (*C*_t_=40), DNA is considered absent. *C*_t_ >36 and <40 represent measured data, but for absolute quantification only values of *C*_t_ ⩽36 were used for reasons of reliability.

### TOPO TA cloning

All cloning was carried out using a TOPO TA Cloning kit (Invitrogen, UK) according to the manufacturers instructions. TOPO TA cloning takes advantage of the nontemplate-dependent terminal transferase activity of *Taq* polymerase that adds a single deoxyadenosine (A) to the 3′ends of PCR products. The linearised vector supplied with the kit has a single, overhanging 3′deoxythymidine (T) residue, allowing efficient ligation between PCR product and vector. The presence of an insert of the correct size was confirmed by *Eco*RI restriction fragment analysis in the vector pCR4-TOPO.

## RESULTS

### Development of a real-time PCR assay for the quantification of the 3895 bp deletion

**Figure 2:**
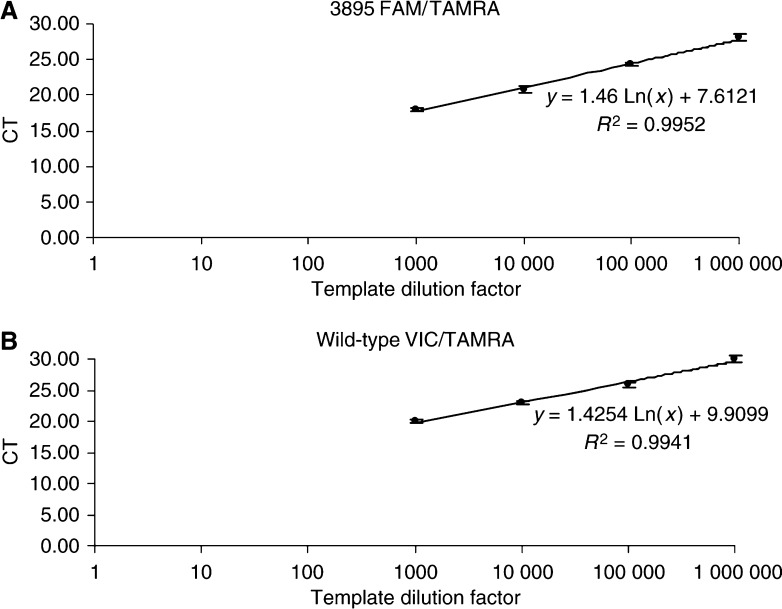
Sensitivity of real-time PCR to template copy number. Threshold cycle (i.e. *C*_t_, vertical axis) at decreasing concentrations of template DNA (horizontal axis) for the 3895 bp deletion (**A**) or wild type internal standard (**B**) are shown. There is a linear relationship between template concentration and the threshold cycle number (*C*_t_) for both amplifications. Each number represents the mean ±s.d. for three independent observations.

We first determined whether it was possible to reliably detect and quantify the percentage of copies of the mitochondrial genome harbouring the 3895 bp mtDNA deletion. We, therefore, examined the linearity of the two PCR reactions using either the internal standard probe (IS-probe, Figure 1) or the 3895 bp deletion probe (3895-probe, Figure 1) over a wide range of template concentrations. In both cases, we generated template DNA by cloning the appropriate PCR product into a cloning vector (see Materials and Methods). The real-time PCR amplifications were performed using serial dilutions of DNA encompassing between 50 ng and 50 pg of template DNA for each probe (Figure 2). The relationship between the *C*_t_ value and the template concentration was linear for both the 3895 bp deletion (*r*=0.9952, Figure 2A) and the internal standard (*r*=0.9941, Figure 2B). In addition, the gradient for amplification of each template was the same for both the 3895 bp deletion and the internal standard cloned templates. Importantly, this confirms that each template is amplified to the same degree of efficiency. As a result, the *C*_t_ values can therefore be used as a measure of template DNA and to quantify the relative amount of 3895 bp deletion compared to wild-type mtDNA. Furthermore, the ability of these standard curves to accurately predict the ratio of deleted: wild-type mtDNA was confirmed by using a range of the cloned deleted : wild-type template mixtures from 0 to 100% (results not shown).

### Quantification of the 3895 bp deletion in tumours

Previous investigations of the 3895 bp deletion in NMSC have been limited to a brief single study of a small number of samples using a standard nonquantitative PCR assay which provides simple data such as the presence or absence of the deletion (Krishnan *et al*, 2004). In our current investigations, we now determine the level of the 3895 bp deletion within human skin taken from NMSCs and the corresponding histologically normal perilesional dermis and epidermis using the developed real-time Taqman PCR assay described above. These results were then compared to the data obtained by analysing the same samples using the previously established standard PCR assay and ethidium bromide stained gels (Krishnan *et al*, 2004).

**Figure 3:**
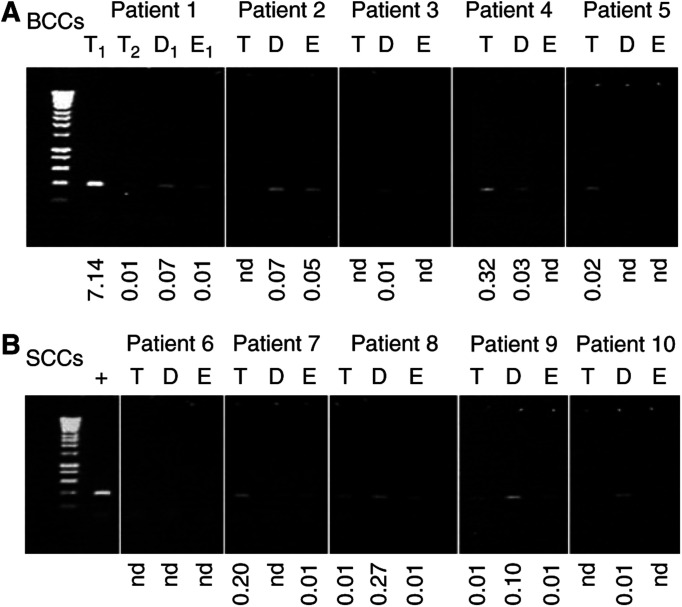
Real-time PCR quantification and standard PCR amplification of the 3895 bp deletion in tumours. Ethidium bromide stained agarose gel showing the incidence of the 3895 bp deletion in tumour (T) and histologically normal perilesional dermis (D) and epidermis (E) from both BCCs (**A**) and SCCs (**B**) using the standard PCR assay. There are two different tumour samples (T_1_ and T_2_) from patient 1 (**A**); the associated dermis and epidermis are taken perilesional to T_1_. Below each lane is shown the level of the 3895 bp deletion illustrated as a percentage in each sample as quantified by real-time taqman PCR. Those samples marked with nd are determined to be effectively zero or ‘not detectable’ as the *C*_t_ of the real-time PCR was >36, which is the level observed in the no template control. Lane 1 in all panels=molecular weight markers (Hyperladder IV – range 1000–100 bp, Bioline Ltd, London UK). The positive (+) control in (**B**) is the tumour DNA from which the PCR product was cloned and sequenced to produce the template for real-time PCR. The same amount of template DNA was added to each PCR reaction. It worth noting that the greater sensitivity of the real-time PCR assay compared to the standard PCR assay is shown by the fact that detectable levels of the deletion are observed with the real-time assay even though there are no detectable PCR amplicons on the ethidium stained gel.

The results of this comparison show that the levels of the 3895 bp deletion quantified by real-time PCR were generally in concordance with those estimated by the standard nonquantitative PCR analysis (Figure 3). The simple pattern of occurrence of the 3895 bp deletion in BCCs was generally similar to that observed in SCCs. In the tumour samples the deletion was present in three of five of both BCC and SCC patients (although not the same patients). In the perilesional skin the deletion tended to occur more frequently in the dermis of the BCC samples (four of five) compared to the epidermis (two of five) although the number of samples was not sufficient for meaningful statistical analysis. However, although the absolute number of samples is small, there is additional valuable information provided by the actual level of the deletion as determined by the real-time PCR assay rather than its simple pattern of occurrence as determined by the standard nonquantitative PCR analysis. For example in both the SCC and BCC patients, the level of the deletion in the dermis was higher than its corresponding epidermal sample. In addition, it was interesting to observe that BCC samples taken from two distinct areas of the face showed vastly different levels of the deletion (i.e. 7.14 *vs* 0.02%), which may reflect variation in the degree of cumulative sun exposure with body site. We decided to investigate this aspect further by determining the level, as opposed to the pattern of occurrence, of the deletion in a relatively large subset of histologically normal perilesional skin samples taken from different sun-exposed human body sites.

### Quantification of the 3895 bp deletion in a larger subset of histologically normal perilesional skin samples taken from different sun-exposed body sites

**Figure 4:**
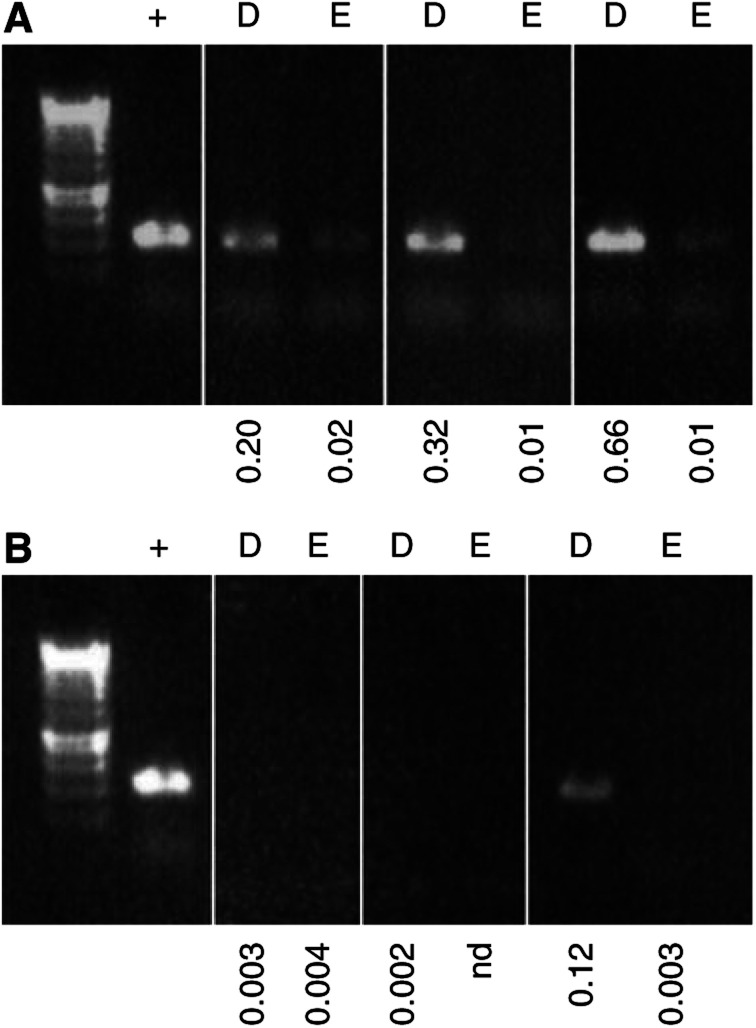
Real-time PCR quantification and standard PCR amplification of the 3895 bp deletion in usually sun-exposed and occasionally sun-exposed skin. Representative ethidium bromide agarose gels showing typical examples of the frequency of occurrence of the 3895 bp deletion as determined by the standard PCR assay in three pairs of usually (**A**) and occasionally (**B**) sun-exposed samples. Below each lane is shown the level of the 3895 bp deletion illustrated as a percentage in each sample as quantified by real-time Taqman PCR. Lane 1 in both panels=molecular weight markers (Hyperladder IV – range 1000–100 bp, Bioline Ltd, London UK). The same amount of template DNA was added to each PCR reaction. The positive control (+) in both panels is the tumour DNA from which the PCR product was cloned and sequenced to produce the template for real-time PCR. Those samples marked with nd are determined to be effectively zero or ‘not detectable’ as the *C*_t_ of the real-time PCR was >36, which is the level observed in the no template control.

**Figure 5:**
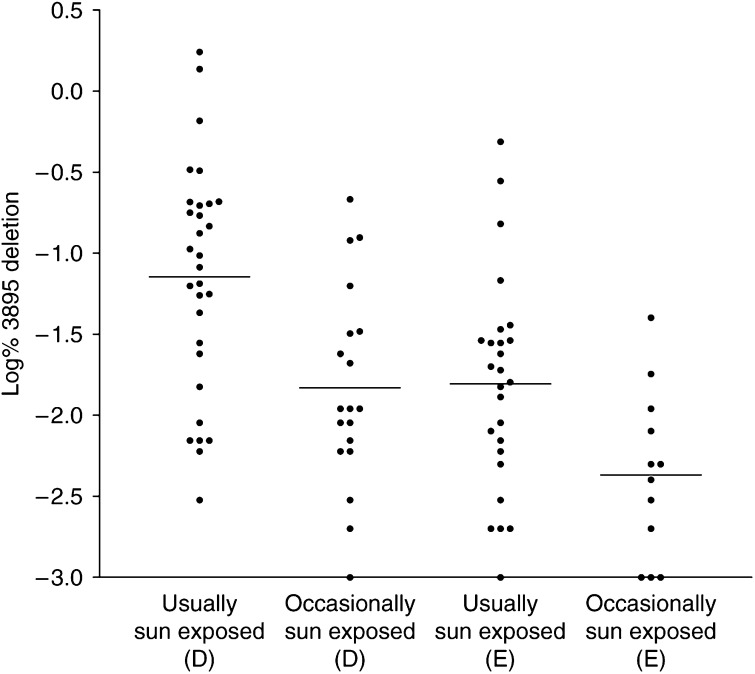
Quantification of the 3895 bp deletion in usually and occasionally sun-exposed human skin. Scatter plot showing the levels of the 3895 bp deletion expressed as a percentage of wild-type mtDNA in usually and occasionally sun-exposed dermis (D) and epidermis (E), as determined by real-time Taqman PCR. The mean level of deletion is indicated by a horizontal line for each set of samples.

Histologically normal perilesional skin, rather than tumour samples were chosen so as to avoid confounding factors other than the site of cumulative sun exposure. We investigated the 3895 bp deletion in 104 age-matched split human skin samples taken from various sun-exposed sites defined as usually exposed (*n*=60) and occasionally exposed (*n*=44) when outdoors (Armstrong, 2004). According to this definition body sites which are ‘usually’ exposed to the sun when outdoors include the scalp, face, neck and ears while those which are ‘occasionally’ exposed to the sun include shoulders, back and chest. Figure 4 shows a representative comparison of results from three pairs of usually and occasionally sun-exposed samples using both the quantitative real-time PCR assay compared with the standard nonquantitative PCR assay (i.e. ethidium bromide stained agarose gel of the 3895 bp amplicon). A comparison of the levels of the 3895 bp deletion detected by real-time PCR with those detected by standard PCR showed once again a good correlation between the two PCR assays. We, therefore, decided to analyse all the samples using the quantitative real-time PCR assay. The results from this analysis (Figure 5) clearly showed an increased incidence of the 3895 bp deletion with increasing sun exposure. In specific terms, the quantitative real-time PCR analysis showed a significantly higher level of the deletion in the usually sun-exposed samples when compared to the occasionally sun-exposed samples (*P*=0.0009 for dermis, *P*=0.008 for epidermis; two-tailed *t*-test). Interestingly, the dermal samples harboured a higher level of the deletion than the epidermis (*P*=0.0143 occasionally exposed, *P*=0.0007 usually exposed).

## DISCUSSION

### The level of the 3895 bp deletion is higher in samples from usually compared to occasionally sun-exposed skin

Previous work by ourselves and others have shown a UVR-associated increase in the incidence of the mtDNA damage in sun-exposed compared to rarely sun-exposed human skin when outdoors. The major limitation of these studies is their failure to address the fact that sun-exposed body sites are divided into two distinct categories, usually (i.e. face and hands) and occasionally (i.e. trunk and legs) (Armstrong, 2004). This is vitally important for skin cancer research as the vast majority of NMSCs occur on usually sun-exposed sites. Previous findings have shown that the frequency of occurrence (i.e. the simple presence or absence) of the 3895 bp deletion is increased with increasing sun exposure (Harbottle *et al*, 2004; Krishnan *et al*, 2004). Our present study has extended this work because it has used a quantitative real-time PCR assay to investigate the actual level of the 3895 bp in different sun-exposed body sites. The results from this analysis clearly showed an increased incidence of the 3895 bp deletion with increasing sun exposure. In specific terms, the quantitative real-time PCR analysis showed a significantly higher level of the deletion in the usually sun-exposed samples when compared to the occasionally sun-exposed samples (*P*=0.0009 for dermis, *P*=0.008 for epidermis; two-tailed *t*-test). The mean ages, sex ratios and tumour type from which the perilesional skin was taken were very similar between the usually sun-exposed and occasionally exposed groups (see Materials and methods) which implies that our findings are unlikely to be confounded by these factors.

### Functional significance

The regions that are deleted in the 3895 bp deletion are from the mtTF1-binding site in the D-loop to tRNA methionine. Deleted genes include 12 s rRNA, 16 s rRNA, ND1 and also the promoters for transcription of both the H and L strands. The 3895 bp deletion levels observed in this study are up to 2% in the sun-exposed skin samples and 7% in the NMSC samples and therefore may have a partial deficiency effect on oxidative phosphorylation. It is known in human muscle that individual fibers can differ in their level of mtDNA deficiency throughout the muscle and that these differing levels will contribute to an overall average of mtDNA deficiency in a given muscle sample (Brierley *et al*, 1998). Therefore, it is more likely that the levels observed in skin represent an overall average and that individual cells and/or focal areas will have several fold higher and lower areas of damage throughout the sample. This may result in a deficiency of oxidative phosphorylation in these focal areas. This scenario is further complicated by the recessive nature of mtDNA (Chomyn *et al*, 1992; Sciacco *et al*, 1994), the threshold of mtDNA damage which is required to cause a biochemical consequence (Porteous *et al*, 1998) and the capacity of mtDNA to harbour a high mutation load. At the tissue rather than the cellular level, these effects can also be dependent on the energetic capacity of the tissue as there is a higher proportion of mitochondria in tissues with high energy demands. The resultant effect in skin, however, is uncertain at present and may be related to the different energetic demands in the differentiating epidermis compared to the dermis.

### Putative mechanism

It has been previously suggested that the mechanism for the generation of the 4977 bp deletion involves a intragenomic recombination event via slipped strand mispairing which may occur at the 13 bp repeats flanking the deletion (Schon *et al*, 1989; Shoffner *et al*, 1989; Mita *et al*, 1990; Degoul *et al*, 1991). This mechanism requires that regions on both the heavy and light mtDNA strands are simultaneously single-stranded. However, neither of the 13 bp repeats are single-stranded in a simultaneous event during replication (Schon *et al*, 1989). Schon *et al*, also suggested that homopyrimidine regions in the 13 bp repeat/or flanking AT-rich regions may be susceptible to DNA bending that would ‘open up’ a small region or ‘bubble’ of single-stranded DNA. Interestingly, these homopyrimidine regions map to structurally labile ‘hot regions’ for the 4977 bp common deletion that assume an unusual bent-DNA structure and may therefore enhance the intragenomic recombination event (Hou and Wei, 1998). The results in our current study and our previous work (Harbottle *et al*, 2004; Krishnan *et al*, 2004) suggest strongly that UVR is a contributing factor in the generation of the 3895 bp deletion. Furthermore, we know that the 3895 bp deletion is flanked by 12 bp repeats. It is therefore tempting to propose that the generation of the 3895 bp deletion may occur by a similar mechanism to the proposed model for the 4977 bp common deletion. Therefore, one might suggest that prolonged UVR exposure could either directly (by inducing base substitutions as opposed to deletions) or indirectly (by induction of free radicals) affect these 12 bp repeat structurally labile sites through opening a ‘bubble’ of single-stranded DNA that would enhance the recombination event, thereby eliciting an increase in mtDNA deletions. In this respect, it is of interest to note that investigation of the 3895 bp deletion in the same NMSC samples (Krishnan *et al*, 2004) which were used in a previous study of the 4977 bp common deletion (Durham *et al*, 2003), showed a comparatively greater frequency of occurrence of the 3895 bp deletion. As these tumours were excised from body sites that are usually exposed to the sun, it is interesting to speculate that the 3895 bp deletion may be a more sensitive marker of cumulative sun exposure than the 4977 bp common deletion.

### The dermis harbours a higher level of the 3895 bp deletion than the epidermis

Our study has also extended previous findings (Birch-Machin *et al*, 1998; Ray *et al*, 2000; Krishnan *et al*, 2004) by showing that the level rather than simply the frequency of occurrence of the 3895 bp deletion is significantly higher in the dermis compared with the epidermis both in the occasionally sun-exposed samples (*P*=0.0143) and in the usually sun-exposed skin (*P*=0.0007). This observation was confirmed by the results of the real-time PCR analysis of the tumours and the corresponding perilesional skin (i.e. dermis and epidermis) from the NMSC patients. Initially, this observation would seem unexpected as the epidermis, being the upper layer of the skin, receives a higher total proportion of UVR and also acts as a filter of UVR for the dermis. However, based on evidence from previous studies in several tissues (Ikebe *et al*, 1990; Corral-Debrinski *et al*, 1991; Lee *et al*, 1994), it has been proposed (Cortopassi *et al*, 1992) that there is often more mtDNA damage (particularly deletions, although the 3895 bp is not specifically mentioned) in tissues that turn over slowly or not at all (e.g. brain and muscle) than in those that turn over relatively more rapidly (e.g. liver and blood cells). Mitochondria harbouring mutant mtDNA will of course suffer a disadvantage during the energy consuming process of replication (Kowald, 1999). This effect will be highest in rapidly dividing cells and so the high turnover of keratinocytes may give little time for accumulation of mtDNA deletions. In contrast the low proliferative rate of fibroblasts may give more opportunity for the accumulation of deleted mtDNA. Furthermore the higher activity of antioxidant enzymes such as superoxide dismutase and catalase in the epidermis compared with the dermis (Shindo *et al*, 1994) would contribute to the decreased mutational load observed in the epidermis. In addition, it worth noting that it is the longer UV wavelengths that penetrate to the dermis and may therefore contribute to the damage possibly through photosensitisation and singlet oxygen production (Birch-Machin, 2006) rather than directly by UV-induced photoproducts such as pyrimidine dimers or six to four photoproducts. The deletion-junction sequence that is characteristic of the 3895 bp deletion is 5′ CTAACC ^536 bp/4430 bp^ccataccccgaa^548 bp/4442^AATGTT 3′. Characteristically this sequence contains only one of the two 12 bp repeats that flank the 3895 bp deletion in wild-type mtDNA (lower case letters). It is clear, however, that the repeat sequence harbours adjacent pyrimidines for possible photoproduct formation. As these repeat regions are structurally labile, it is possible that the deletion occurs as a result of a combination of direct induction of photoproducts coupled with oxidative stress, the relative contribution of these effects may be associated with skin depth and hence UVR penetration.

### Concluding remarks

In summary, our present study has extended previous work by developing and then using a quantitative real-time PCR assay to investigate for the first time the actual level (as opposed to the freqeuncy of occurrence) of the 3895 bp deletion in human skin from different sun-exposed body sites and tumours from NMSC patients. The results from this analysis clearly show an increased level of the 3895 bp deletion with increasing sun exposure. In specific terms, the quantitative real-time PCR analysis showed a significantly higher level of the deletion in the usually sun-exposed samples when compared to the occasionally sun-exposed skin. Our study has also extended previous findings by showing that the level rather than simply the frequency of occurrence of the 3895 bp deletion is significantly higher in the dermis compared with the epidermis both in the occasionally sun-exposed and in the usually sun-exposed skin samples. The determination of the actual level of the 3895 bp deletion provides an opportunity to investigate several research areas. For example, it provides a method for studying the association between the actual level of the mutational load and tumour progression including the involvement of precursor lesions. In addition, through the use of laser capture microscopy it would be possible to determine the actual level of mtDNA damage within focal areas found in different layers of the skin. As a final example, a reliable real-time PCR assay for the 3895 bp deletion will provide a means of comparing the relative amounts of the 3895 bp and 4977 bp common mtDNA deletion within the same skin sample and help to provide some clues as to the aetiology of the two types of mtDNA damage in relation to sun exposure.

## Figures and Tables

**Table 1:** Oligonucleotide primers used to detect the 3895 bp deletion in the standard and real-time PCR assays

**Name**	**Dye**	**Position**	**Sequence**
ISF		16042-16066	5′-GAT TTG GGT ACC ACC CAA GTA TTG-3′
ISR		16125-16102	5′-AAT ATT CAT GGT GGC TGG CAG TA-3′
IS-Probe	Vic	16069-16101	5′-CAC CCA TCA ACA ACC GCT ATG TAT TTC GTA CA-3′Tamra
3895F		491-508	5′-CAA CCC TCG CCC ATC CTA-3′
3895R		4516-4489	5′-CCT GCA AAG ATG GTA GAG TAG ATG AC-3′
3895-probe	Fam	527//4450	5′-TGC TAA CCC CAT ACC CCG AAA ATG TTG G-3′Tamra
L404		404-423	5′-CTT TTG GCG GTA TGC ACT TT-3′
H4676		4676-4657	5′-GAT TAT GGA TGC GGT TGC TT-3′

## References

[bib1] Armstrong BK (2004) How sun exposure causes skin cancer: an epidemiological perspective. In Prevention of Skin Cancer, Hill D, Elwood JM, English DJ (eds) Vol. 3. pp. 89–116. Cancer Prevention – Cancer Causes. Drodrecht: Kluwer Acedemic Publishers

[bib2] Berneburg M, Gattermann N, Stege H, Grewe M, Vogelsang K, Ruzicka T, Krutmann J (1997) Chronically ultraviolet-exposed human skin shows a higher mutation frequency of mitochondrial DNA as compared to unexposed skin and the hematopoietic system. Photochem Photobiol 66: 271–275 9277148 10.1111/j.1751-1097.1997.tb08654.x

[bib3] Berneburg M, Grether-Beck S, Kurten V, Ruzicka T, Briviba K, Sies H, Krutmann J (1999) Singlet oxygen mediates the UVA-induced generation of the photoaging-associated mitochondrial common deletion. J Biol Chem 274: 15345–15349 10336420 10.1074/jbc.274.22.15345

[bib4] Birch-Machin MA (2000) Mitochondria and skin disease. Clin Exp Dermatol 25: 141–146 10733641 10.1046/j.1365-2230.2000.00605.x

[bib5] Birch-Machin MA (2006) The role of mitochondria in ageing and carcinogenesis. Clin Exp Dermatol 31: 1–5 16716161 10.1111/j.1365-2230.2006.02161.x

[bib6] Birch-Machin MA, Tindall M, Turner R, Haldane F, Rees JL (1998) Mitochondrial DNA deletions in human skin reflect photo- rather than chronologic aging. J Invest Dermatol 110: 149–152 9457910 10.1046/j.1523-1747.1998.00099.x

[bib7] Brierley EJ, Johnson MA, Lightowlers RN, James OF, Turnbull DM (1998) Role of mitochondrial DNA mutations in human aging: implications for the central nervous system and muscle. Ann Neurol 43: 217–223 9485063 10.1002/ana.410430212

[bib8] Chomyn A, Martinuzzi A, Yoneda M, Daga A, Hurko O, Johns D, Lai ST, Nonaka I, Angelini C, Attardi G (1992) MELAS mutation in mtDNA binding site for transcription termination factor causes defects in protein synthesis and in respiration but no change in levels of upstream and downstream mature transcripts. Proc Natl Acad Sci USA 89: 4221–4225 1584755 10.1073/pnas.89.10.4221 PMC49053

[bib9] Clayton DA, Doda JN, Friedberg EC (1974) The absence of a pyrimidine dimer repair mechanism in mammalian mitochondria. Proc Natl Acad Sci USA 71: 2777–2781 4212385 10.1073/pnas.71.7.2777 PMC388554

[bib10] Corral-Debrinski M, Stepien G, Shoffner JM, Lott MT, Kanter K, Wallace DC (1991) Hypoxemia is associated with mitochondrial DNA damage and gene induction. Implications for cardiac disease. JAMA 266: 1812–1816 1890710

[bib11] Cortopassi GA, Shibata D, Soong NW, Arnheim N (1992) A pattern of accumulation of a somatic deletion of mitochondrial DNA in aging human tissues. Proc Natl Acad Sci USA 89: 7370–7374 1502147 10.1073/pnas.89.16.7370 PMC49711

[bib12] Croteau DL, Bohr VA (1997) Repair of oxidative damage to nuclear and mitochondrial DNA in mammalian cells. J Biol Chem 272: 25409–25412 9325246 10.1074/jbc.272.41.25409

[bib13] Czarnecka AM, Golik P, Bartnik E (2006) Mitochondrial DNA mutations in human neoplasia. J Appl Genet 47(1): 67–78 16424612 10.1007/BF03194602

[bib14] Degoul F, Nelson I, Amselem S, Romero N, Obermaier-Kusser B, Ponsot G, Marsac C, Lestienne P (1991) Different mechanisms inferred from sequences of human mitochondrial DNA deletions in ocular myopathies. Nucleic Acids Res 19: 493–496 2011523 10.1093/nar/19.3.493 PMC333638

[bib15] Durham S, Betts J, Birch-machin M (2002) Histochemical and mutational analysis of mitochondrial function and DNA in tumours and matched normal human skin. Brit J Derm 146: 733–734

[bib16] Durham SE, Krishnan KJ, Betts J, Birch-Machin MA (2003) Mitochondrial DNA damage in non-melanoma skin cancer. Br J Cancer 88: 90–95 12556965 10.1038/sj.bjc.6600773 PMC2376793

[bib17] Harbottle A, Krishnan KJ, Birch-Machin MA (2004) Implications of using the ND1 gene as a control region for real-time PCR analysis of mitochondrial DNA deletions in human skin. J Invest Dermatol 122: 1518–1521 15175045 10.1111/j.0022-202X.2004.22608.x

[bib18] Hou JH, Wei YH (1998) AT-rich sequences flanking the 5′-end breakpoint of the 4977-bp deletion of human mitochondrial DNA are located between two bent-inducing DNA sequences that assume distorted structure in organello. Mutat Res 403: 75–84 9726008 10.1016/s0027-5107(98)00054-2

[bib19] Ikebe S, Tanaka M, Ohno K, Sato W, Hattori K, Kondo T, Mizuno Y, Ozawa T (1990) Increase of deleted mitochondrial DNA in the striatum in Parkinson's disease and senescence. Biochem Biophys Res Commun 170: 1044–1048 2390073 10.1016/0006-291x(90)90497-b

[bib20] Koch H, Wittern KP, Bergemann J (2001) In human keratinocytes the Common Deletion reflects donor variabilities rather than chronologic aging and can be induced by ultraviolet A irradiation. J Invest Dermatol 117: 892–897 11676829 10.1046/j.0022-202x.2001.01513.x

[bib21] Kowald A (1999) The mitochondrial theory of aging: do damaged mito-chondria accumulate by delayed degradation? Exp Gerontol 34: 605–612 10530786 10.1016/s0531-5565(99)00011-x

[bib22] Krishnan KJ, Harbottle A, Birch-Machin MA (2004) The use of a 3895 bp mitochondrial DNA deletion as a marker for sunlight exposure in human skin. J Invest Dermatol 123: 1020–1024 15610508 10.1111/j.0022-202X.2004.23457.x

[bib23] LeDoux SP, Patton NJ, Avery LJ, Wilson GL (1993) Repair of *N*-methylpurines in the mitochondrial DNA of xeroderma pigmentosum complementation group D cells. Carcinogenesis 14: 913–917 8504484 10.1093/carcin/14.5.913

[bib24] Lee HC, Pang CY, Hsu HS, Wei YH (1994) Differential accumulations of 4,977 bp deletion in mitochondrial DNA of various tissues in human ageing. Biochim Biophys Acta 1226: 37–43 8155737 10.1016/0925-4439(94)90056-6

[bib25] Mita S, Rizzuto R, Moraes CT, Shanske S, Arnaudo E, Fabrizi GM, Koga Y, DiMauro S, Schon EA (1990) Recombination via flanking direct repeats is a major cause of large-scale deletions of human mitochondrial DNA. Nucleic Acids Res 18: 561–567 2308845 10.1093/nar/18.3.561 PMC333462

[bib26] Pang CY, Lee HC, Yang JH, Wei YH (1994) Human skin mitochondrial DNA deletions associated with light exposure. Arch Biochem Biophys 312: 534–538 8037468 10.1006/abbi.1994.1342

[bib27] Parr RL, Dakubo GD, Thayer RE, McKenney K, Birch-Machin MA (2006) Mitochondrial DNA as a potential tool for early cancer detection. Hum Genom 2(4): 252–257 10.1186/1479-7364-2-4-252 PMC3500203 16460650

[bib28] Pascucci B, Versteegh A, van Hoffen A, van Zeeland AA, Mullenders LH, Dogliotti E (1997) DNA repair of UV photoproducts and mutagenesis in human mitochondrial DNA. J Mol Biol 273: 417–427 9344749 10.1006/jmbi.1997.1268

[bib29] Porteous W, James A, Sheard P, Porteous C, Packer M, Hyslop S, Melton J, Pang C, Wei Y, Murphy P (1998) Bioenergetic consequences of accumulating the common 4977-bp mitochondrial DNA deletion. Eur J Biochem 257: 192–201 9799119 10.1046/j.1432-1327.1998.2570192.x

[bib30] Ray AJ, Turner R, Nikaido O, Rees JL, Birch-Machin MA (2000) The spectrum of mitochondrial DNA deletions is a ubiquitous marker of ultraviolet radiation exposure in human skin. J Invest Dermatol 115: 674–679 10998142 10.1046/j.1523-1747.2000.00092.x

[bib31] Sawyer DE, Van Houten B (1999) Repair of DNA damage in mitochondria. Mutat Res 434: 161–176 10486590 10.1016/s0921-8777(99)00027-0

[bib32] Schon EA, Rizzuto R, Moraes CT, Nakase H, Zeviani M, DiMauro S (1989) A direct repeat is a hotspot for large-scale deletion of human mitochondrial DNA. Science 244: 346–349 2711184 10.1126/science.2711184

[bib33] Sciacco M, Bonilla E, Schon EA, DiMauro S, Moraes CT (1994) Distribution of wild-type and common deletion forms of mtDNA in normal and respiration-deficient muscle fibers from patients with mitochondrial myopathy. Hum Mol Genet 3: 13–19 8162014 10.1093/hmg/3.1.13

[bib34] Severi G, English DJ (2004) Descriptive epidemiology of skin cancer. In Prevention of Skin Cancer, Hill D, Elwood JM, English DJ (eds), Vol. 3. pp 73–88. Cancer Prevention – Cancer Causes. Dordrecht: Kluwer Acedemic Publishers

[bib35] Shindo Y, Witt E, Han D, Epstein W, Packer L (1994) Enzymic and non-enzymic antioxidants in epidermis and dermis of human skin. J Invest Dermatol 102: 122–124 8288904 10.1111/1523-1747.ep12371744

[bib36] Shoffner JM, Lott MT, Voljavec AS, Soueidan SA, Costigan DA, Wallace DC (1989) Spontaneous Kearns-Sayre/chronic external ophthalmoplegia plus syndrome associated with a mitochondrial DNA deletion: a slip-replication model and metabolic therapy. Proc Natl Acad Sci USA 86: 7952–7956 2554297 10.1073/pnas.86.20.7952 PMC298190

[bib37] Wesson KM, Silverberg NB (2003) Sun protection education in the United States: what we know and what needs to be taught. Cutis 71: 71–74, 77 12553634

